# Spaced training improves learning in Ts65Dn and *Ube3a* mouse models of intellectual disabilities

**DOI:** 10.1038/s41398-019-0495-5

**Published:** 2019-06-10

**Authors:** J. C. Lauterborn, M. N. Schultz, A. A. Le, M. Amani, A. E. Friedman, P. T. Leach, C. M. Gall, G. S. Lynch, J. N. Crawley

**Affiliations:** 1 Department of Anatomy & Neurobiology, School of Medicine, University of California Irvine, Irvine, CA 92697 USA; 2 MIND Institute, Department of Psychiatry and Behavioral Sciences, University of California Davis School of Medicine, Sacramento, CA 95817, USA; 3 Department of Psychiatry and Human Behavior, School of Medicine, University of California Irvine, Irvine, CA 92697 USA; 4 Department of Physiology, School of Medicine, Ardabil University of Medical Sciences, Ardabil, Iran; 5 Present Address: Harvard University, Cambridge, MA, USA; 6 Present Address: Biogen Inc., Cambridge, MA USA

**Keywords:** Learning and memory, Psychiatric disorders

## Abstract

Benefits of distributed learning strategies have been extensively described in the human literature, but minimally investigated in intellectual disability syndromes. We tested the hypothesis that training trials spaced apart in time could improve learning in two distinct genetic mouse models of neurodevelopmental disorders characterized by intellectual impairments. As compared to training with massed trials, spaced training significantly improved learning in both the Ts65Dn trisomy mouse model of Down syndrome and the maternally inherited *Ube3a* mutant mouse model of Angelman syndrome. Spacing the training trials at 1 h intervals accelerated acquisition of three cognitive tasks by Ts65Dn mice: (1) object location memory, (2) novel object recognition, (3) water maze spatial learning. Further, (4) spaced training improved water maze spatial learning by *Ube3a* mice. In contrast, (5) cerebellar-mediated rotarod motor learning was not improved by spaced training. Corroborations in three assays, conducted in two model systems, replicated within and across two laboratories, confirm the strength of the findings. Our results indicate strong translational relevance of a behavioral intervention strategy for improving the standard of care in treating the learning difficulties that are characteristic and clinically intractable features of many neurodevelopmental disorders.

## Introduction

As first recognized in the late 19th century^1^, and subsequently confirmed by a very large body of human studies^2–10^, multiple training episodes spaced apart in time produce better learning than a single massed session. This spaced trials or distributed practice effect is ubiquitous in that it is observed in many species and across a very broad range of learning paradigms^11–28^. Psychologists have advanced several ingenious theories to explain this fundamental aspect of learning, each of which has received experimental support. One of the most widely discussed of these hypotheses begins with environmental changes over time and posits that spacing results in the association of core information with multiple contexts. This results in a greater number of retrieval cues and a reduced likelihood that core elements will become associated with transient aspects of the environment^29–32^. An alternative and also highly regarded hypothesis is based on the evidence that memories gradually stabilize with time. These retrieval theories argue that spacing is effective because successive sampling periods add to partially consolidated memory traces^33–35^. An important variant of this idea proposes that distributed learning allows for rehearsal of newly consolidated memory, something that would not happen with massed sessions because the original memory traces are still active^4^. It is possible that no single mechanism accounts for the extreme diversity of spaced trials effects and that the above proposals, with their multiple variants, apply to different aspects of the phenomenon.

While little is known about the possible contributions of synaptic plasticity rules to the efficacy of spacing, recent work on LTP in hippocampal field CA1 has provided evidence potentially related to consolidation models. Specifically, attempts to produce additional potentiation were unsuccessful when applied at 10–40 min after the initial induction of LTP but doubled the magnitude of the effect when delayed by 50–60 min^21^. Other experiments found that a previously undetected second stage of consolidation for LTP emerges after the same interval^36^. Mechanisms underlying both the enhanced LTP and the delayed stabilization were described^37^. Work using a type of learning that is dependent on field CA1 then confirmed the behavioral relevance of the LTP results^36^. Whether these effects occur at other forebrain sites possibly with different timing rules has yet to be tested.

Given the ubiquity of the spaced trials effect, and some evidence relating to substrates, it is surprising that little attention has been given to the possibility of using the paradigm to improve learning in various neurodevelopmental disorders with intellectual disabilities. There is however a report suggesting that cue sampling at one-hour intervals significantly reduces the learning impairment found in the *Fmr1*-KO model of Fragile-X syndrome^38^. The present studies tested the generalizability, reproducibility, and robustness of temporally spaced training trials as an intervention for cognitive impairments. We evaluated learning after massed versus spaced training in two genetically distinct mouse models of intellectual disability: (1) the Ts65Dn trisomy model of Down syndrome^39–55^, and (2) the *Ube3a* maternally-derived knockout model of Angelman syndrome^56–64^. Two laboratories independently tested mice from separate colonies, including comparisons of cohorts bred in-house versus purchased commercially. The generality of any spaced trial benefits across diverse cognitive assays^65^ was evaluated using four learning and memory tasks, with different sensory and motor requirements and different neuroanatomical substrates. Our laboratories^38,60,64,66^ and others cited above had previously demonstrated that these tests detect significant impairments in mouse models of neurodevelopmental disorders including Down and Angelman syndromes. In all, a combination of animal models, testing sites, and behavioral paradigms was used to strengthen conclusions about the potential benefits of spaced training for offsetting cognitive problems associated with aberrant brain development.

## Materials and methods

### Mice

All studies were approved by the University of California Irvine and University of California Davis Institutional Animal Care and Use Committees, using procedures consistent with the NIH Guide for the Care and Use of Laboratory Animals. Mice were purchased from The Jackson Laboratory and bred as described below. At both facilities, housing cages were maintained in AAALAC-approved temperature and humidity controlled vivaria on a conventional 12:12 light cycle, with lights on at 7 AM and behavioral testing conducted during the light phase of the circadian cycle, between 8 AM and 5 PM. Food and water were provided ad libitum. Mice were 7–14 weeks of age during testing. Object location memory in male WT and Ts65Dn mice was conducted at UC Irvine. Novel object recognition, open field activity, rotarod, and Morris water maze were conducted at UC Davis, in that sequence, for male and female WT and Ts65Dn mice. Male and female WT and *Ube3a* mice were tested at UC Davis in the sequence of open field, rotarod, water maze. Data from males and females were combined in the statistical analyses. Low numbers of males and females per group precluded detection of sex differences. Larger Ns will be needed in future studies to draw rigorous conclusions about potential sex differences in performance between male and female mice of each genotype in these assays.

### Breeding

To generate in-house bred subject mice for the Down syndrome model, female Ts65Dn (JAX #005252, which do not harbor the retinal degeneration gene), were mated with males of the same background strain, B6eiC3F1 (JAX #003647), as previously described^64^. To generate in-house bred subject mice for the Angelman syndrome model, in which the mutation is maternally derived, heterozygous female *Ube3a* mice (JAX catalogue #016590) were mated with males of the same background strain, C57BL/6J (JAX #000664), as previously described^64^. Tailsnips were genotyped by TransnetYX (Cordova, TN) for UC Davis studies, or using Jackson Laboratory PCR methods and KAPA2G HS DNA polymerase (Thermo Fisher Scientific #NC0562625) for UC Irvine studies. Offspring were weaned at 21–25 days of age into cages of 2–4 mice of mixed genotypes, housed by sex. Males and females of each genotype from each litter and post-weaning housing cage were randomly assigned to training condition groups, to reduce potential effects of any differences in maternal care and home cage environments. Coded identification numbers ensured that investigators remained blind to genotype during testing. For both Ts65Dn and *Ube3a* lines at UC Davis, Cohort 1 was composed of mice purchased from JAX and Cohort 2 was composed of mice bred in-house. Data are presented separately for each of the two Ts65Dn cohorts in the results, to display the similarities in findings from externally purchased versus vivarium-bred mice.

### Behavioral assays

Four widely studied behavioral paradigms with varying sensory and motor requirements and different neuroanatomical substrates were used in the present studies. These included (1) object location memory^38,66–72^, (2) novel object recognition^54,70,71,73–84^, (3) Morris water maze spatial learning^44,62,64,69,76,77,85–89^, and (4) rotarod motor learning^62,64,78,87,90–101^.

#### Object location memory

The object location memory test was conducted using methods previously described^38,66^. Experiments were conducted in a darkened room with overhead lighting (235 lux) directly above the testing chambers. On Days 1–4, all mice were handled for 10 min to acclimate them to the investigator and to being removed from their home cage. On Days 5–10, each mouse was habituated to an empty white testing chamber (30 × 24 cm floor; 30 cm high) for 5 min per day. Familiarization training occurred on Day 11. For massed training, the subject mouse was placed into the chamber with two identical objects (small glass funnels) located along the same wall, each within ~3 cm of an arena corner. The mouse was allowed to freely explore during the 10 min training session. 24 h later, the mouse was reintroduced to the chamber with one object in the original familiar location and the other object placed in the diagonal corner (novel location). During this test phase for object location memory, the mouse was allowed to explore for 5 min, then returned to its home cage. For spaced training, the mouse was similarly placed into the chamber with two identical objects each located along the same wall, each within ~3 cm of an arena corner. Three familiarization training trials were administered, each 3.3 min long, with 1 h intervals between trials. The subject mouse was returned to its home cage between trials. 24 h later, the mouse was tested for object location memory during a 5 min exploration period of the chamber with one of the objects moved to the novel location. All objects and chambers were cleaned following training and testing using 1X SCOE and dried. Sessions were recorded by an overhead video camera. Locomotor activity was analyzed with Noldus Ethovision software. Time (t) spent in exploratory sniffing of each object was quantified offline from the videotapes by raters who were blind to genotype and training treatment. Mice were scored for time spent exploring each object, when the nose was touching or within 0.5 cm of the object. Grooming, passing by, or head orientation in another direction were excluded. Total time spent in object exploration was quantified as the combined time interacting with both objects. To assess preferential attention to an object, a discrimination index was calculated as 100 × (t_novel_ **−** t_familiar_) ÷ (t_novel_ + t_familiar_), with a positive discrimination index representing preference for the novel location.

#### Novel object recognition

The novel object recognition test for episodic recognition memory was conducted using methods previously described^71,102^, except for the spaced training protocol described below. On Day 1, each mouse was placed in an empty white plastic testing chamber (40 × 40 cm) and allowed to explore for 30 min to habituate to the arena. On Day 2, the subject mouse was placed in the same empty arena for a second habituation session of 30 min. On day 3, the subject mouse was placed in the same empty arena for a third habituation session of 10 min. The mouse was removed, and two identical objects were placed in the chamber ~12 cm from the wall and ~18 cm apart (familiarization session). Objects used were either two orange cones (Amazon.com), or two green cylindrical magnets (Magneatos, Guide Craft, Amazon.com). For massed training, the mouse was replaced into the arena and allowed to explore the two identical objects for 10 min. After this familiarization session, for massed testing, the subject mouse was removed from the arena and returned to its home cage. For spaced training, the mouse was replaced into the arena with the two identical objects and allowed to explore the test arena for 3.3 min, placed in a holding cage in another room for 1 h, placed back in the arena with the two identical objects for another 3.3 min, returned to the holding cage for 1 h, placed back in the arena with the two identical objects for 3.3 min, and returned to its home cage. After the familiarization sessions, objects were cleaned with a weak Alconox detergent solution, and chambers were cleaned with 70% ethanol. On day 4, 24 h after the end of familiarization in both conditions, one identical object and one novel object, i.e., cone and cylinder, were placed into the arena in the same locations. The subject mouse was returned to the arena and allowed to explore both objects for 5 min. Novel object recognition was defined as spending significantly more time sniffing the new object than sniffing the familiar object. The novel objects, cone and cylinder, previously determined to elicit similar amounts of sniffing in control mice, were counterbalanced across subject mice to further prevent object bias. Exploratory activity and time spent sniffing each object were scored by Noldus Ethovision XT software (Wageningen, The Netherlands), using three body point identification to include only sniffing directed toward and within 2 cm of the object. Discrimination index was calculated as 100 × (t_novel_ **−** t_familiar_) ÷ (t_novel_ + t_familiar_), with a positive discrimination index representing preference for the novel object.

#### Open field exploratory activity

Open field locomotion was scored during the 30 min habituation session on Day 2, as an internal control for general exploratory activity. Open field parameters were automatically videotracked and quantified by Noldus Ethovision XT. Parameters of total distance, horizontal activity, vertical activity, and center time were collected in 5 min time bins and summed for the full session length of 30 min.

#### Rotarod

Rotarod motor coordination and balance was tested using an Ugo Basile accelerating mouse rotarod, (Stoelting Co., Wood Dale, Illinois) as previously described^64^. Revolutions per minute (rpm) were set at an initial value of 5 rpm, increasing progressively to a maximum of 40 rpm across 5 min. Massed training consisted of 3 trials, with 60 sec intervals between each trial. Spaced training consisted of 3 trials, with 1 h intervals between each trial, consistent with the timing employed in the other three cognitive tasks. Latency to fall was automatically detected by the equipment and recorded for each trial.

#### Water maze

Mice were trained in the hidden platform version of the Morris water maze using methods consistent with standards in the literature and as previously described^64,77^. A 120 cm circular pool was filled with water (24–25 °C). Crayola liquid non-toxic white paint was added for opacity, to prevent proximal visual detection of the hidden platform. External cues for distal spatial navigation included a computer, large sink, water temperature regulator with yellow hose, a large black X on one wall, a black and white poster on another wall, and a paper lantern hung from the ceiling. Platform locations and start locations were pseudorandomized. Trials were videorecorded and scored by automated software (Noldus Ethovision XT) for measures including latency to find the hidden platform, total distance traveled, and swim speed. For massed training, each subject mouse was given 4 consecutive trials per day, for 10 days, until the WT control group reached the latency criterion of 15 sec or less to reach the hidden platform. Mice were allowed to remain on the platform for ~15 s after each trial. After the fourth daily trial, each mouse was placed under an infrared heating lamp to help restore body temperature. For spaced training, each subject mouse was given 4 trials per day with 1 h between each trial, for 10 days, until the WT control group reached the latency criterion of 15 sec or less to reach the hidden platform. Pilot studies with other daily training regimens had indicated lack of beneficial effects of spaced trials in standard C57BL/6J mice given 2 sets of 2 consecutive trials separated by a 1 h interval between sets, or 3 trials separated by 1 h intervals, as compared to 4 continuous massed trials (unpublished studies by Adam Friedman and Prescott Leach). Four daily water maze training trials separated by 1 h intervals appeared to offer an optimal paradigm to specifically evaluate the effects of spaced training in mouse models of intellectual disabilities. After each training trial, subject mice were allowed to remain on the platform for ~15 s, then placed under an infrared heating lamp to help restore body temperature. Probe trial analysis, to confirm that learning the hidden platform location was accomplished using distal environmental room cues and to evaluate long-term memory of the location of the hidden platform, was conducted at 24 h after the last training trial. Duration of each probe trial was 60 s. Time spent in each of the four pool quadrants, and number of crossings over the former platform location versus the three analogous imaginary platform locations in the other quadrants, were automatically scored by the Noldus videotracking software.

### Statistical analyses

Object location memory and novel object recognition data were analyzed (a) with paired t-tests, comparing the number of seconds spent sniffing each object within genotype and within training condition, during the familiarization and novel object recognition phases, and (b) with a discrimination index (DI, defined as (seconds spent with novel minus seconds spent with familiar) divided by total time (novel + familiar), as previously described^70^. Locomotor activity associated with the object location memory test was analyzed using One-Way Analysis of Variance followed by Tukey’s multiple comparisons post-hoc test in cases of significant ANOVA F values. Open field parameters were compared between genotypes using One-Way Analysis of Variance (ANOVA), followed by Tukey’s posthoc in cases of a significant ANOVA F value. Rotarod data were analyzed with Two-Way ANOVA using genotype as a between subjects factor and trial as a within subjects factor. Water maze acquisition parameters were evaluated with a Two-Way Repeated Measures ANOVA followed by Bonferroni posthoc tests in cases of significant ANOVA *F* values. Water maze probe trial data were evaluated with One-Way ANOVA followed by post-hoc Dunnett’s multiple comparisons tests to compare the target location to the other three locations, within genotype and within training condition. Data were analyzed and graphed with GraphPad Prism version 7.

## Results

**Fig. 1:**
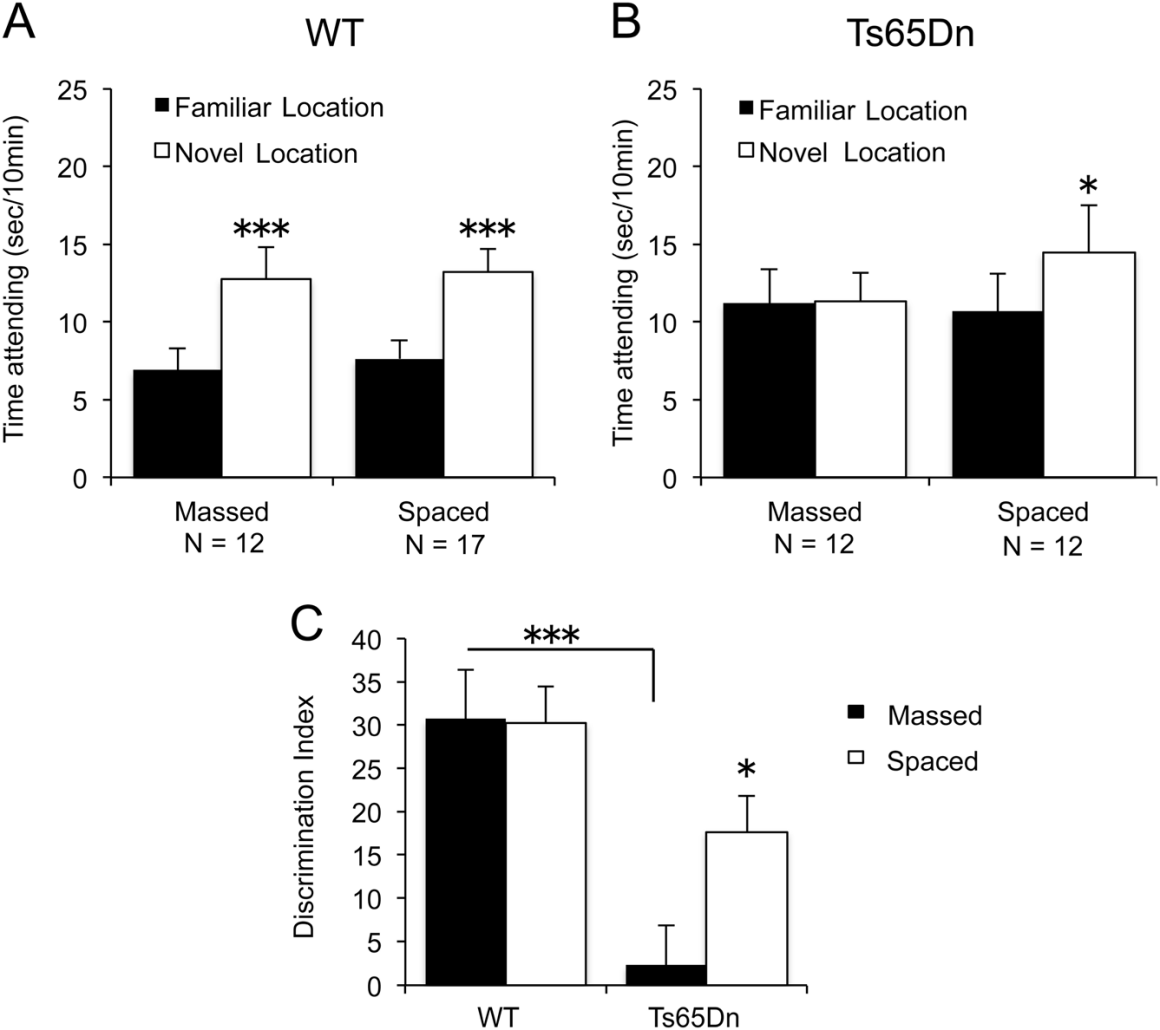
Object location memory in WT and Ts65Dn mice. Object location memory was detected in WT for both the massed and spaced training conditions. Ts65Dn did not display object location memory in the massed training condition, whereas the spaced training condition yielded significant object location memory. **a** WT displayed significantly more time exploring the object in the novel location versus the object in the familiar location, both when training trials were administered consecutively (massed: *t*_1,11_ = 4.66, ***p < 0.001), and when the three training trials were administered at 1 h intervals (spaced: *t*_1,16_ = 6.57, ***p < 0.001). No effect of training condition alone or of the interaction between training condition and object locations was detected in WT. **b** Ts65Dn did not display a significant difference between time spent exploring the object in the familiar location and the object in the novel location when training trials were administered consecutively (massed: *t*_1,11_ = 0.109, NS). Ts65Dn displayed significantly more time exploring the object in the novel location versus the object in the familiar location when training trials were administered at 1 h intervals (spaced: *t*_1,11_ = 3.06, df_1,11_, *p < 0.02). A significant interaction between training conditions and object locations was detected by Two-Way ANOVA for Ts65Dn (*p* < 0.05). **c** Discrimination index (DI) was lower in Ts65Dn mice given massed training trials as compared to WT given massed training trials (***p < 0.001). Spaced training trials significantly elevated the DI in Ts65Dn, as compared to the DI in Ts65Dn given massed training trials (*p < 0.05). A significant interaction between genotype and training condition was detected by Two-Way ANOVA (*p* < 0.05). In all figures, data are expressed as mean + standard error of the mean. Numbers of mice in each genotype and training condition group are displayed within the graphs

Figure 1 summarizes performance in the object location memory (OLM) test for the Ts65Dn mouse model of Down syndrome and WT littermate controls in experiments conducted at UC Irvine. Past studies showed that WT animals acquire memory in this test after a five or ten minute session of unsupervised exposure to the cues^103^. We used a 10-min session to maximize massed trial performance by the mutants. As anticipated, the WT controls had a pronounced preference for the novel location in tests conducted 24 h after a single massed training period. Spaced exposures to the cues on day one did not increase this high retention score (Fig. 1a, see legends for statistical results). As predicted, Ts65Dn failed to display OLM, showing equivalent numbers of seconds spent exploring the object in the new location and the object in the original location during the retention trial when the familiarization training trials were massed in one 10-min session. In contrast, when familiarization was spaced into 3 training trials, each of 3.3 min duration, separated by 1-h intervals, Ts65Dn mice displayed significant OLM (Fig. 1b). Data analysis using the derived discrimination index confirmed that supra-threshold massed training produced no evidence for long-term memory in Ts65Dn, whereas this striking defect was partially prevented by spaced training (Fig. 1c).

The encoding problem found in the Ts65Dn animals during massed training could not be attributed to a failure in cue sampling, since both genotypes spent the same total amount of time exploring the objects during the session and as well in the subsequent retention test (Supplementary Fig. S1a). This was also the case for each of the three spaced trials and the delayed memory measurement (Fig. S1b). Overall exploration of the apparatus during habituation and massed training was also comparable between the groups (Fig. S1c). This also held for exploration during the three spaced training sessions (Fig. S1d). Finally, total arena exploration time did not differ between learning protocols or genotypes (Fig. S1e). We conclude from these sampling times and activity measurements that the Ts65Dn mutation did not affect the manner in which the animals interact with simple cues or explore a simple environment, confirming that object location memory scores were not confounded by exploration artifacts.

**Fig. 2:**
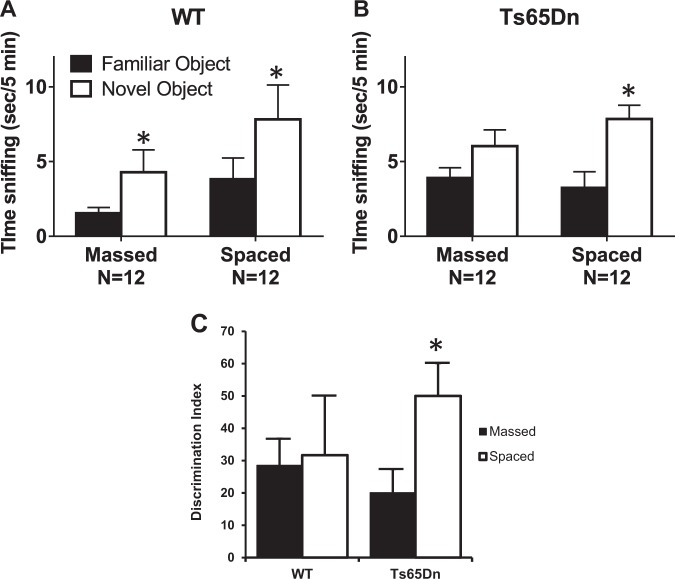
Novel object recognition in WT and Ts65Dn mice. Novel object recognition memory was detected in WT for both the massed and spaced training conditions. Ts65Dn did not display novel object recognition in the massed training condition, whereas the spaced training condition yielded significant novel object recognition. **a** WT displayed significantly more time exploring the novel object than the familiar object, both when training trials were administered consecutively (massed: *t*_1,11_ = 2.29, *p < 0.05), and when the three training trials were administered at 1 h intervals (spaced: *t*_1,10_ = 2.31, *p < 0.05). **b** Ts65Dn did not display a significant difference between time spent exploring the novel object and time spent exploring the familiar object (massed: *t*_1,11_ = 2.035, NS). Ts65Dn displayed significantly more time exploring the novel object than the familiar object when training trials were administered at 1 h intervals (spaced: *t*_1,11_ = 3.60, *p < 0.01). **c** Spaced training trials significantly elevated the DI in Ts65Dn, as compared to the DI in Ts65Dn given massed training trials (*p < 0.05). Interaction between genotype and training condition was not significant

Figure 2 shows analogous results in Ts65Dn mice tested on novel object recognition, a second recognition learning and memory assay, conducted at UC Davis. We again used an unsupervised sampling period (10 min) that from past reports^38^ is supra-threshold for learning cue identity in WT mice. Controls spent more time exploring the novel object than the familiar object after both massed and spaced training (Fig. 2a). Ts65Dn showed equivalent numbers of seconds spent sniffing the novel object and the familiar object when the familiarization training trials were massed in one 10 min session, indicating failure to acquire or remember the object properties. In contrast, when familiarization was spaced into 3 training trials separated by 1-hour intervals, Ts65Dn mice displayed a marked and significant novel object recognition effect (Fig. 2b). Data analyses using the derived discrimination index confirmed that spaced training profoundly enhanced object learning in the mutants (Fig. 2c). As shown in Supplementary Fig. 2, sampling times for the two cues during the acquisition session did not differ between massed and spaced trials for the WTs (Fig. S2a) or mutants (Fig. S2b). There was a tendency for the latter to spend more time with the objects but this did not reach statistical significance. As with the OLM paradigm, amount of general arena exploration did not differ between genotypes (Fig. S2c).

**Fig. 3:**
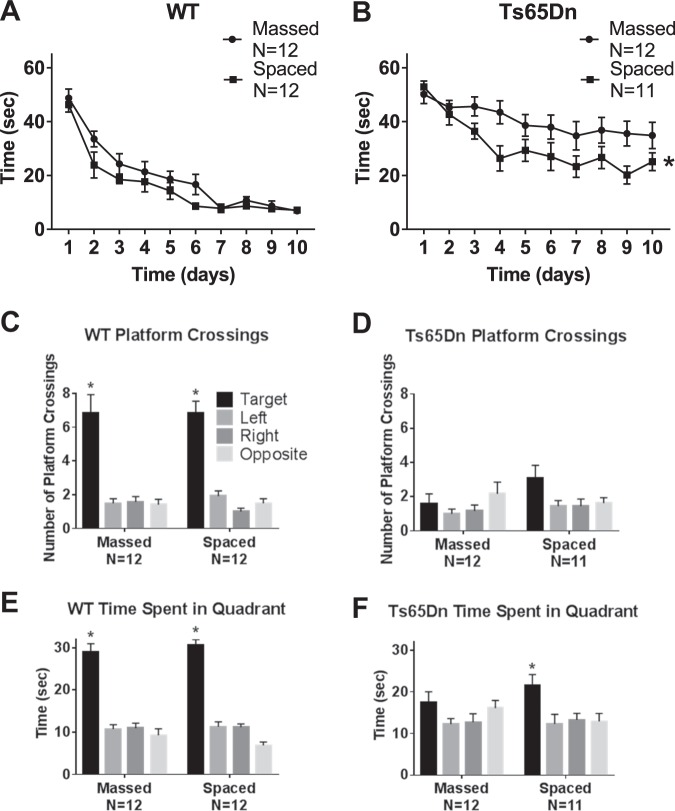
Morris water maze performance in WT and Ts65Dn mice, Cohort 1. Ts65Dn displayed impaired spatial learning, which was improved by training with distributed trials spaced at 1 h intervals. **a** WT successfully achieved the acquisition criterion of 15 s or less to reach the hidden platform location. No significant difference was detected in the time course for acquisition by WT mice trained with massed versus spaced trials (Two-Way ANOVA *F*_1,23_ = 0.101, NS), indicating no faster learning in WT trained with spaced trials. A significant effect of training day was detected in WT (*F*_9,207_ = 18.97, *p* < 0.001), indicating learning across days as expected. No significant interaction between massed versus spaced x training day was detected in WT (*F*_9,207_ = 0.410, NS). **b** Ts65Dn trained with massed trials did not achieve the acquisition criterion of 15 s or less to reach the hidden platform location. A significant difference was detected in the time course for acquisition by Ts65Dn mice trained with massed versus spaced trials (*F*_1,24_ = 8.064, *p < 0.001), indicating faster learning with spaced training trials. A significant effect of training day was detected (*F*_9,216_ = 5.99, *p* < 0.001), indicating some learning across training days. No significant interaction between massed versus spaced x training day was detected in Ts65Dn (*F*_9,216_ = 1.02, NS). **c-f** Probe trial performance 24 h after the last training trial. **c** WT crossed the previously trained target platform location significantly more times than over the corresponding left, right, and opposite locations, in both the massed and spaced training conditions (massed: *F*_3,44_ = 19.52, *p < 0.001; Dunnett’s multiple comparisons adjusted *p* values: target vs. left *p* < 0.001, target vs. right *p* < 0.001, target vs. opposite p < 0.001; spaced: *F*_3,44_ = 41.33, *p < 0.001; target vs. left *p* < 0.001, target vs. right *p* < 0.0001, target vs. opposite *p* < 0.0001). **d** Ts65Dn did not cross the previously trained target platform location significantly more than the corresponding left, right, and opposite locations, in either the massed or spaced training conditions, although a trend was seen after spaced training (massed: *F*_3,44 = _1.096, NS; spaced: *F*_3,40_ = 2.739, *p* = 0.06, NS). **e** WT spent more time in the previously trained target quadrant than in the left, right, and opposite quadrants, for both massed and spaced training groups (massed: *F*_3,44_ = 41.72, *p < 0.001; target vs. left *p* < 0.001, target vs. right *p* < 0.001, target vs. opposite *p* < 0.001; spaced: *F*_3,44_ = 104.6, *p < 0.001; target vs. left *p* < 0.001, target vs. right *p* < 0.001, target vs. opposite *p* < 0.001). **f** Ts65Dn spent significantly more time in the previously trained target quadrant than in the left, right, and opposite quadrants after spaced training but not in the massed training condition (massed: *F*_3,44_ = 1.597, NS; spaced: *F*_3,40_ = 4.289, *p < 0.01; target vs. left *p* < 0.01, target vs. right *p* < 0.05, target vs. opposite *p* < 0.05)

Figure 3 illustrates the deficits in water maze learning in Ts65Dn mice, and presents evidence that these deficits were ameliorated by spaced training trials. Data in Fig. 3 represent one full cohort of WT and Ts65Dn mice, purchased from JAX and housed at UC Davis. WT reached the criterion of ≤15 s to reach the hidden platform after seven days of training, both when the four training trials were massed consecutively and when the four trials were separated by 1 h intervals. Ts65Dn failed to reach criterion in both conditions, but performance, defined as reduction in time to locate the hidden platform across training days, was significantly better when the four training trials were spaced by 1 h intervals rather than massed. In the spaced training condition, Ts65Dn displayed decreasing latencies during the first 4 days of training, but no further improvement across days 5–10. In contrast, WT in the spaced training condition displayed latencies which decreased through training day 7, and remain below criterion through day 10. These results suggest that mice with the Ts65Dn mutation have some capacity for spatial learning, particularly when training trials are temporally spaced, but may not have abilities sufficient to reach the same performance asymptote as controls, at least in the present paradigm. Probe trial analysis 24 h after the last training trial confirmed that WT mice had learned the hidden platform location using distal spatial room cues and remembered the hidden platform location one day later. WT made more crossings over the previous platform location than the corresponding locations in each of the other three quadrants, and spent more time swimming in the quadrant that previously contained the hidden platform than in the other three quadrants. These results were obtained in both the massed and spaced training conditions. Ts65Dn did not make significantly more crossings over the previous platform location in either training condition. Equivocal findings were obtained for time spent in the trained quadrant. Ts65Dn in the spaced training group spent significantly more time swimming in the previously trained quadrant than in the other three quadrants, while the massed training group did not. However, number of seconds spent in the trained quadrant was very similar in Ts65Dn trained with massed and spaced trials. Supplementary Fig. 3a,b confirms that initial swim speeds were similar between genotypes, indicating normal motor swimming abilities in Ts65Dn mice. The acquisition curve results support the interpretation that spaced training improved spatial learning in Ts65Dn mice, although performance levels reached by WT were not fully achieved by Ts65Dn.

**Fig. 4:**
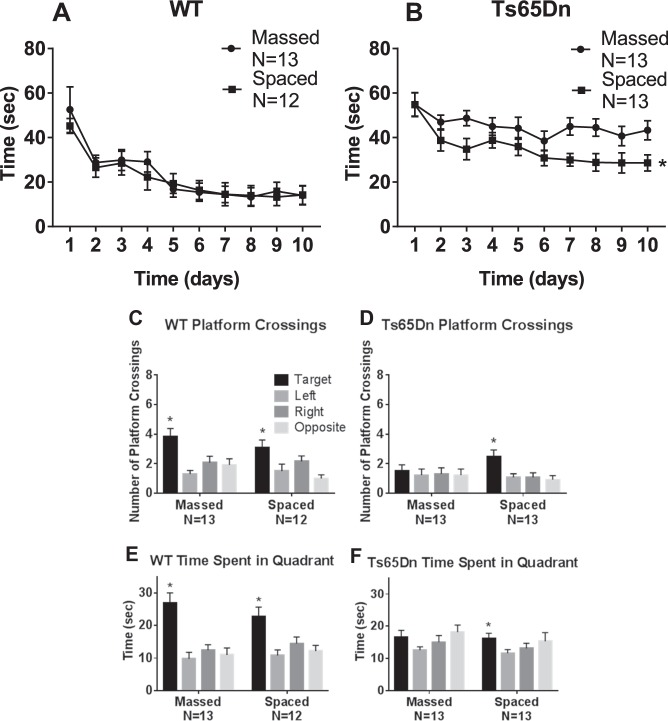
Morris water maze performance in WT and Ts65Dn mice, Cohort 2 replication. As seen in Cohort 1, a second independent cohort of Ts65Dn similarly displayed impaired spatial learning which was improved by training with distributed trials spaced at 1 h intervals. **a** WT successfully achieved the acquisition criterion of 15 s or less to reach the hidden platform location. No significance was detected in the time course for acquisition by WT mice trained with massed versus spaced trials (*F*_1,23_ = 0.110, NS), indicating no faster learning in WT trained with spaced trials. A significant effect of training day was detected in WT (*F*_9,207_ = 19.0, *p* < 0.001), indicating learning across days as expected. No significant interaction between massed versus spaced x training day was detected in WT (*F*_9,207_ = 0.410, NS). **b** Ts65Dn did not achieve the acquisition criteria of 15 s or less to reach the hidden platform location. A significant difference was detected in the time course for acquisition by Ts65Dn mice trained with massed versus spaced trials (*F*_1,24_ = 8.06, *p < 0.01), indicating faster learning with spaced training trials. A significant effect of training day was detected (*F*_9,216_ = 5.60, *p* < 0.001), indicating some learning across training days. No significant interaction between massed versus spaced x training day was detected in Ts65Dn (*F*_9,216_ = 1.02, NS). Three-way ANOVA detected significance for latency (*F*_1,9_ = 23.1, *p* < 0.001), genotype (*F*_1,1_ = 35.8, *p* < 0.001), no significance for latency x treatment (*F*_1,9_ = 0.295, NS), or latency x genotype x treatment (*F*_1,9_ = 1.11, NS). **c–f** Probe trial performance 24 h after the last training trial. **c** WT crossed the previously trained target platform location significantly more than over the corresponding left, right, and opposite locations, in both the massed and spaced training conditions (massed: *F*_3,48_ = 6.85, *p < 0.001; Dunnett’s multiple comparisons adjusted *p* values: target vs. left *p* < 0.001, target vs. right *p* < 0.05, target vs. opposite *p* < 0.01; spaced: *F*_3,44_ = 4.681, *p < 0.01; target vs. left *p* < 0.05, target vs. opposite *p* < 0.01. **d** Ts65Dn crossed the previously trained target platform location significantly more than the corresponding left, right, and opposite locations, after spaced training but not after massed training trials (massed: *F*_3,48_ = 0.129, NS, spaced: *F*_3,48_ = 4.64, *p < 0.01; target vs. left *p* < 0.05, target vs. right *p* < 0.05, target vs. opposite *p* < 0.01). **e** WT spent more time in the previously trained target quadrant than in the left, right, and opposite quadrants, for both massed versus spaced training groups (massed: *F*_3,48_ = 12.5, *p < 0.001; target vs. left *p* < 0.001, target vs. right *p* < 0.001, target vs. opposite *p* < 0.001; spaced: *F*_3,44_ = 6.45, *p < 0.001; target vs. left *p* < 0.001, target vs. right *p* < 0.05, target vs. opposite *p* < 0.01). **f** Ts65Dn spent more time in the previously trained target quadrant than in the left, right, and opposite quadrants, after spaced training but not after massed training trials (massed: *F*_3,48_ = 2.32, NS; spaced: *F*_3,48_ = 3.39, *p < 0.05; target vs. left *p* < 0.05, target vs. right *p* < 0.05)

Figure 4 summarizes water maze learning results in a second cohort of Ts65Dn mice, which was bred in-house at UC Davis. Again, WT reached criterion both in the massed and spaced training conditions. Ts65Dn failed to reach criterion in either condition, but performance was significantly better when the four training trials were spaced by 1 h intervals as compared to massed. Probe trial analysis 24 h after the last training trial confirmed that WT had learned and remembered the hidden platform location, on measures of platform location crossings and quadrant time, in both the massed and spaced training conditions. Ts65Dn that received massed training failed to display significantly more crossings over the previous platform location, and did not spend more time in the previously trained quadrant in the massed condition. Ts65Dn that received spaced training showed significantly more crossings over the previous platform location and significantly more time swimming in the previously trained quadrant. However, number of seconds spent in the trained quadrant was similar in Ts65Dn after massed and spaced training. Supplementary Fig. 3c,d confirms that initial swim speeds were similar between genotypes, indicating normal motor swimming abilities in the second cohort of Ts65Dn mice. These acquisition curves in cohort 2 corroborate the interpretation that spaced training improved spatial learning in Ts65Dn mice, although WT performance levels were not reached. Replicated findings in two full cohorts, one purchased directly from JAX and one bred in-house, confirm the strength of the water maze results.

**Fig. 5:**
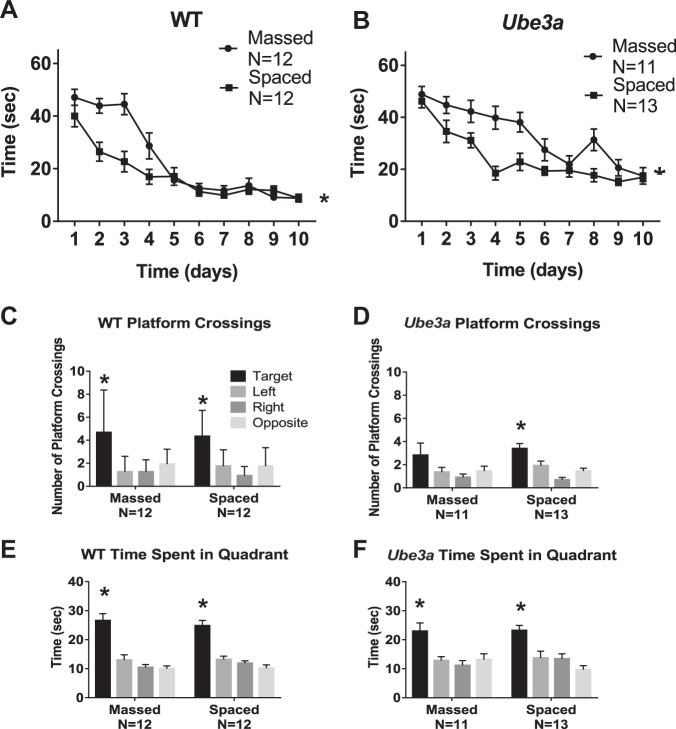
Morris water maze performance in WT and *Ube3a* mice. *Ube3a* displayed impaired spatial learning, which was improved by training with distributed trials spaced at 1 h intervals. **a** WT successfully achieved the acquisition criterion of 15 s or less to reach the hidden platform location. A significant difference was detected in the time course for acquisition by WT mice trained with massed versus spaced trials (*F*_1,22_ = 8.47, *p < 0.01), indicating faster learning in WT trained with spaced trials. A significant effect of training day was detected in WT (*F*_9,198_ = 49.3, *p* < 0.001), indicating learning across days as expected. A significant interaction between massed versus spaced x training day was detected in WT, (*F*_9,198_ = 5.62, *p* < 0.001). **b** *Ube3a* did not achieve the acquisition criterion of 15 s or less to reach the hidden platform location. A significant difference was detected in the time course for acquisition by *Ube3a* mice trained with massed versus spaced trials (*F*_1,22_ = 15.8, *p < 0.001), indicating faster learning with spaced training trials. A significant effect of training day was detected (*F*_9,198_ = 24.6, *p* < 0.01), indicating some learning across training days. A significant interaction between massed versus spaced x training day was detected in *Ube3a* (*F*_9,198_ = 2.65, *p* < 0.01). Three-way ANOVA detected significance for latency (*F*_1,9_ = 68.9, *p* < 0.001, latency x training condition: *F*_1,9_ = 5.16, *p* < 0.001, latency x genotype x training condition: *F*_1,9 _= 2.74, *p* < 0.01). **c–e** Probe trial performance 24 h after the last training trial. **c** WT crossed the previously trained target platform location significantly more times than over the corresponding left, right, and opposite locations, in both the massed and spaced training conditions (massed: *F*_3,44_ = 6.92, *p < 0.001; Dunnett’s multiple comparisons adjusted *p* values: target vs. left *p* < 0.001, target vs. right *p* < 0.001, target vs. opposite *p* < 0.01; spaced: *F*_3,44_ = 10.2, *p < 0.001, Dunnett’s multiple comparisons adjusted *p* values: target vs. left *p* < 0.001, target vs. right *p* < 0.001, target vs. opposite p < 0.001. **d** *Ube3a* crossed the previously trained target platform location significantly more than the left, right, and opposite corresponding platform locations after spaced training trials, but not after massed training trials (massed: *F*_4,42_ = 1.69, NS; spaced: *F*_3,48_ = 11.1, *p < 0.001; target vs. left *p* < 0.01, target vs. right *p* < 0.001, target vs. opposite *p* < 0.001). **e** WT spent significantly more time in the previously trained target quadrant than in the left, right, and opposite quadrants, for both massed versus spaced (massed: *F*_3,44 _= 22.3, *p < 0.001; target vs. left *p* < 0.001, target vs. right *p* < 0.001, target vs. opposite *p* < 0.001; spaced: *F*_3,44_ = 26.4, *p < 0.001; target vs. left *p* < 0.001, target vs. right *p* < 0.001, target vs. opposite *p* < 0.001). **f** *Ube3a* spent significantly more time in the previously trained target quadrant than in the left, right, and opposite quadrants, for both massed versus spaced (massed: *F*_3,40_ = 6.73, *p < 0.001; target vs. left *p* < 0.01, target vs. right *p* < 0.001, target vs. opposite *p* < 0.01; spaced: *F*_3,48_ = 9.74, *p < 0.001; target vs. left *p* < 0.01, target vs. right *p* < 0.01, target vs. opposite *p* < 0.001)

Next, we tested whether the training paradigm results described above extend to a mouse model for a different neurodevelopmental disorder, Angelman syndrome. Figure 5 describes the deficits in water maze learning in *Ube3a* mice and improvements by spaced training trials. WT reached criterion both in the massed and spaced training conditions. *Ube3a* failed to reach criterion in either condition, but performance was significantly better when the four training trials were spaced by 1 h intervals than when massed consecutively. Probe trial analysis 24 h after the last training trial confirmed that WT had learned the platform location using distal spatial room cues and remembered the former platform location, on measures of platform location crossings and quadrant time, in both the massed and spaced training conditions. *Ube3a* failed to display significantly more crossings over the previous platform location in the massed training condition, but achieved significance in the spaced training condition. *Ube3a* spent significantly more time swimming in the previously trained quadrant in both the massed and spaced conditions, with approximately the same numbers of seconds spent in the trained quadrant for both conditions. Supplementary Fig. 3e,f displays impaired swim speeds in *Ube3a* as compared to WT in the massed group. Higher swim speeds in the *Ube3a* spaced group were seen during the initial training days, raising the possibility of motor improvement as an alternate explanation for the learning curve. However, the magnitude of swim speed improvement was relatively small. Data in Fig. 5 represent one full cohort of WT and *Ube3a* mice purchased from JAX and tested at UC Davis. Poor in-house breeding success of *Ube3a* at UC Davis prevented the generation of a second full cohort for corroborative testing.

**Fig. 6:**
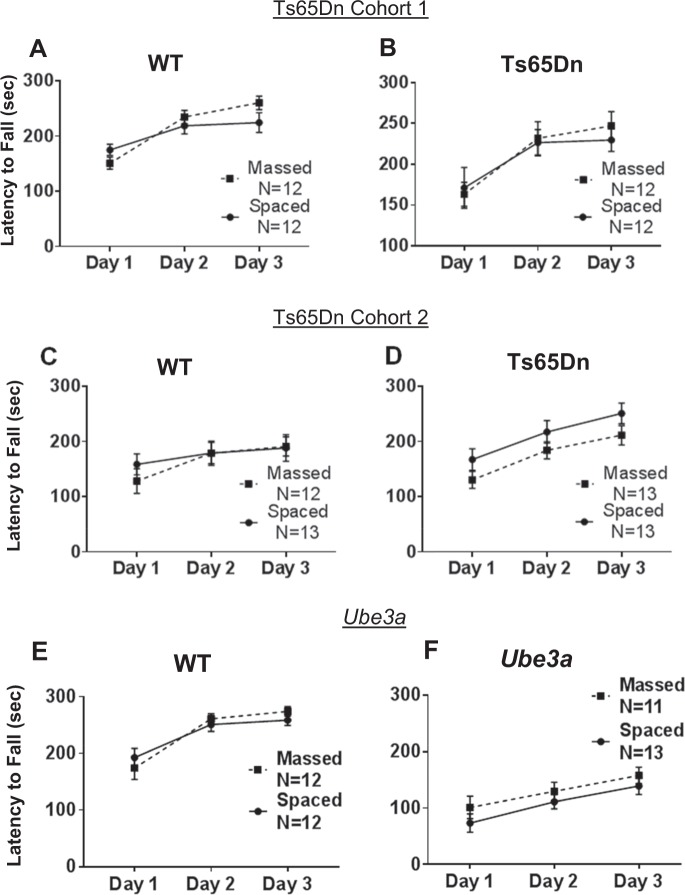
Rotarod motor learning was unaffected by training condition in either Ts65Dn or *Ube3a* mice. **a–d** Ts65Dn mice were not significantly impaired on rotarod performance, as measured by latency to fall from the accelerating rotarod, when compared to WT controls (Cohort 1: *F*_1,1_ = 0.0037, NS; Cohort 2: *F*_1,1_ = 1.57, NS). **a** Cohort 1 WT displayed similar motor learning curves when trained with massed versus spaced trials (*F*_1,22_ = 0.336, NS). WT showed a significant effect of training day (*F*_2,44_ = 45.8, *p* < 0.0001), and a significant interaction between massed versus spaced x training day (*F*_2,44 _= 6.03, *p* < 0.01). **b** Cohort 1 Ts65Dn displayed similar motor learning curves when trained with massed versus spaced trials (*F*_1,22_ = 0.05, NS). Ts65Dn showed a significant effect of training day (*F*_2,44_ = 23.2, *p* < 0.001), but no interaction between massed versus spaced x training day (*F*_2,44_ = 0.613, NS). **c** Cohort 2 WT displayed similar motor learning curves when trained with massed versus spaced trials (*F*_1,23_ = 0.112, NS). WT showed a significant effect of training day (*F*_2,46_ = 15.6, *p* < 0.001), but no interaction between massed versus spaced x training day (*F*_2,46_ = 2.24, NS). **d** Cohort 2 Ts65Dn displayed similar motor learning curves when trained with massed versus spaced trials (*F*_1,24_ = 2.30, NS). Ts65Dn showed a significant effect of training day (*F*_2,48_ = 84.1, *p* < 0.001), but no interaction between massed versus spaced x training day (*F*_2,48_ = 0.116, NS). **e–f** *Ube3*a mice were significantly impaired on rotarod motor learning as compared to WT controls (*F*_1,1 _= 118.6, *p* < 0.001). **e** WT displayed similar motor learning curves when trained with massed versus spaced trials (*F*_1,22_ = 0.044, NS). WT showed a significant effect of training day (*F*_2,44_ = 25.2, *p* < 0.001), but no interaction between massed versus spaced x training day (*F*_2,44_ = 1.02, NS). **f** *Ube3a* displayed similar motor learning curves when trained with massed versus spaced trials (*F*_1,22_ = 1.47, NS). *Ube3a* showed a significant effect of training day (*F*_2,44_ = 11.8, *p* < 0.001), but no interaction between massed versus spaced x training day (*F*_2,44_ = 0.0874, NS)

Figure 6 shows that rotarod motor learning in Ts65Dn and *Ube3a* mice was similar when the three daily training trials were massed consecutively or separated by 1 h intervals. Ts65Dn of both cohorts showed no significant deficits, displaying rotarod performance that was not different than WT controls, and not significantly different between training conditions. *Ube3a* displayed impaired rotarod performance as previously reported^42,62,64^, seen as shorter latencies to fall from the accelerating rotating rod as compared to WT. Spaced training did not significantly improve rotarod performance as compared to massed training in *Ube3a*.

## Discussion

The present studies investigated the possibility that spaced training can be used as a general strategy for treating the learning problems that are a characteristic and clinically intractable feature of many neurodevelopmental disorders (NDD). A useful therapy would have to be applicable to a number of these conditions and, equally important, have beneficial effects across multiple commonplace forms of learning. The findings reported here satisfy these criteria. Spacing training improved learning on tests for encoding of both semantic (cue identity) and spatial information in two mutant mouse models, and in paradigms that did or did not include strong motivation. Notably, although improved performance did not reach fully normal levels on spatial learning, the improvements in recognition and spatial learning tasks occurred against a background of severe learning impairments in animals trained with massed trials. The results were also robust in that they obtained in different laboratories and across separate cohorts of mice. Relatedly, a prior study found marked improvements with spacing in a mouse model for a third neurodevelopmental disorder, Fragile X syndrome^38^, strengthening generalizability across mouse models of three neurodevelopmental disorders with intellectual disabilities.

What types of mechanisms could be responsible for these observations, or more specifically, how does the spacing protocol offset NDD-related disturbances to brain systems for acquiring and utilizing information? Imaging studies in children and adults with these disorders have revealed altered functional connectivity in brain activation patterns, unusual density of white matter tracts, altered cortical thickness, microencephaly and macroencephaly, depending on the genetic mutation^104–108^. Unusual patterns of neuronal dendritic spine morphology have been reported in human postmortem analyses^109–112^. A large number of studies detected analogous abnormalities in dendritic spine morphology in mutant mouse models of neurodevelopmental disorders with intellectual disabilities^43,50,78,111,113–120^. As might be expected from spine aberrations, substantial impairments in the memory-related LTP effect are multiply reported for Ts65Dn, *Ube3a*, and *Fmr1*-KO mice^103,117–122^. In each of these cases, significant progress has been made in identifying defects in the signaling cascades responsible for the stabilization of synaptic potentiation (Ts65Dn:^123–125^, *Ube3a*:^60,126,127^, *Fmr1*-KO:^119,122,128,129^). Moreover, and of considerable interest in the present context, various experimental pharmacological interventions are reported to reduce the magnitude of the LTP deficit and the accompanying learning problems^48,59,60,121,130–140^. In all, while it is unlikely that spacing affects the profound morphological disturbances that characterize the NDD brain, it is possible that the protocol in some manner compensates for defects in the complex machinery that produces plasticity.

Consistent with the above argument, a recent study found that *Fmr1* knockout mice fail to activate an LTP-critical enzyme at hippocampal synapses when given a single learning episode and that this signaling deficit is reduced with trials separated by the minimal interval (1 h) for the “LTP spaced trial” effect^38^. We therefore propose that (i) many NDDs cause breaks in the sequences that consolidate one trial LTP and learning, as described above, and (ii) the events that produce secondary potentiation after a delay are sufficiently intact to produce a net increase in memory-related synaptic strength. The latter part of this argument is testable with procedures used to describe the cell biological substrates for the delayed LTP effect^21^.

While spacing was effective in three of the behavioral paradigms, it produced no evident reductions in the impairments to motor learning on the accelerating rotarod. We found no difference between three massed training trials on each of three training days versus three training trials spaced at one-hour intervals on each of three training days. *Ube3a* displayed its previously reported deficit on rotarod performance, which was not improved by spaced training. Rotarod motor learning is mediated primarily by the cerebellum^87,141–143^, while acquisition in the water maze^144–147^, object location^68,148–150^, and novel object^67,68,151,152^ recognition paradigms are heavily dependent upon structures in the cortical telencephalon. It is reasonable to expect that memory encoding substrates may differ significantly between cerebellar vs. forebrain networks; if so, then the between trials delay used here may not have been appropriate for eliciting spacing effects of the type previously reported for motor learning^5,153^. In any event, the absence of effects in the rotarod task is consistent with the idea that the positive results for spatial and semantic memory reflect activation of LTP-related processes described for hippocampus.

Finally, the present results suggest opportunities for potential clinical applications. Direct comparisons of massed versus spaced training sessions have not been published in the human literature for either Down syndrome or Angelman syndrome. A small number of studies have been published for children with genetically unspecified intellectual impairments. This sparse literature reported better performance after spaced training trials for a transfer task^154^ and better performance after spaced trials on the initial phase of learning a motor skill in one study^155^, although no effect of spaced sessions on motor learning was seen in three other studies^156–158^, consistent with our findings of no differences on rotarod motor learning between massed versus spaced training regimens in Ts65Dn and *Ube3a* mice. In small studies of autism spectrum disorder, spaced practice sessions were more effective than massed practice sessions on syllable learning in three children with autism spectrum disorder^159^, while massed training was more effective than spaced on a pictorial task in six children with autism^160^. It will be interesting to investigate spaced versus massed learning approaches across a range of cognitive tests in children, adolescents and adults with various genetically defined intellectual disability syndromes^137,161–168^. The present findings in the Ts65Dn trisomy mouse model of Down syndrome and the *Ube3a* maternally-derived mutant mouse model of Angelman syndrome, along with our previous parallel findings in the *Fmr1* mutant mouse model of Fragile X syndrome^38^, support the strategy of therapeutic behavioral interventions using spaced sessions of distributed learning opportunities, to enhance cognitive abilities in neurodevelopmental disorders characterized by intellectual disabilities.

## Supplementary information

Supplementary Figures 1-3
