# Supplementary material for: Spaced training improves learning in Ts65Dn and Ube3a mouse models of intellectual disabilities
Source: Transl Psychiatry. 2019 Jun 10;9:166. doi: 10.1038/s41398-019-0495-5 (PMC6557858; doi:10.1038/s41398-019-0495-5)
Supplement: Supplementary file 1 — Supplementary Figures 1-3 [file 41398_2019_495_MOESM1_ESM.pdf]

Supplementary Figure 1

Ts65Dn Object Location Memory Exploration Times and Locomotion

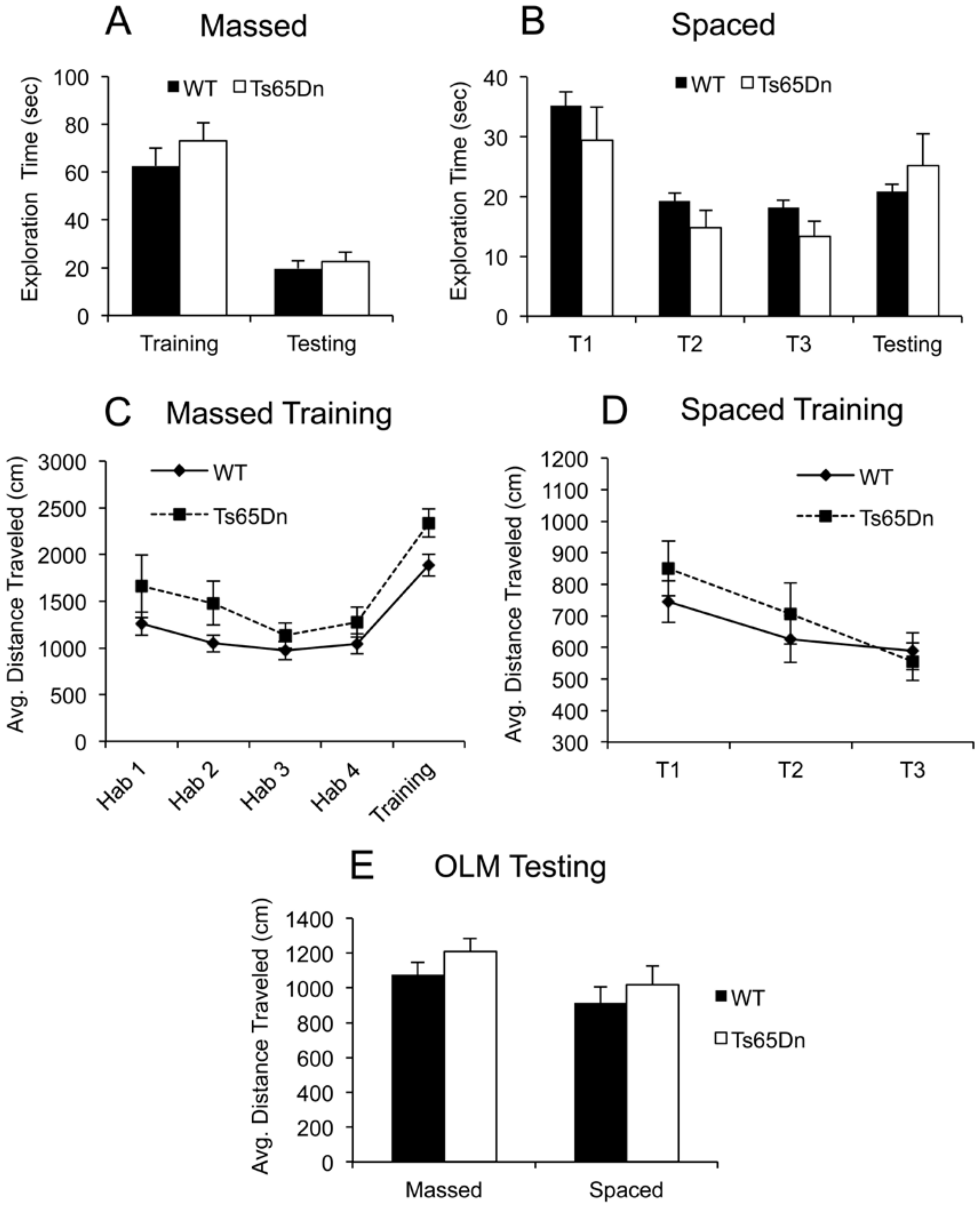

Supplementary Figure 1. Exploratory behaviors associated with object location memory were similar in WT and Ts65Dn mice, indicating no genotype differences in general locomotion or exploratory tendencies. (A) Total exploration times of both objects were similar between genotypes during both the 10 minute massed training session and the test session (training $t_{1,22}$ = 0.968, NS; testing $t_{1,22}$ = 0.559, NS). (B) Total exploration times of the objects were similar between genotypes during each of the three spaced training trials (T1, T2, T3, each 3.3 minutes) and during the subsequent test session (training p = 0.9567, NS) and for interaction (Two-Way ANOVA training trials NS, test $t_{1,27}$ = 0.793, NS). (C) Distance traveled by each genotype was not significantly different during the prior habituation sessions (Hab 1-4) or during the massed training session for either genotype or for interaction between genotype and trial. (D) Distance traveled by each genotype was not significantly different during any of the spaced training trials. (E) Distance traveled did not differ between genotypes during the object location memory testing session in either training condition (massed: $t_{1,22}$ = 1.291, NS; spaced: $t_{1,27}$ = 0.7317, NS).

Supplementary Figure 2

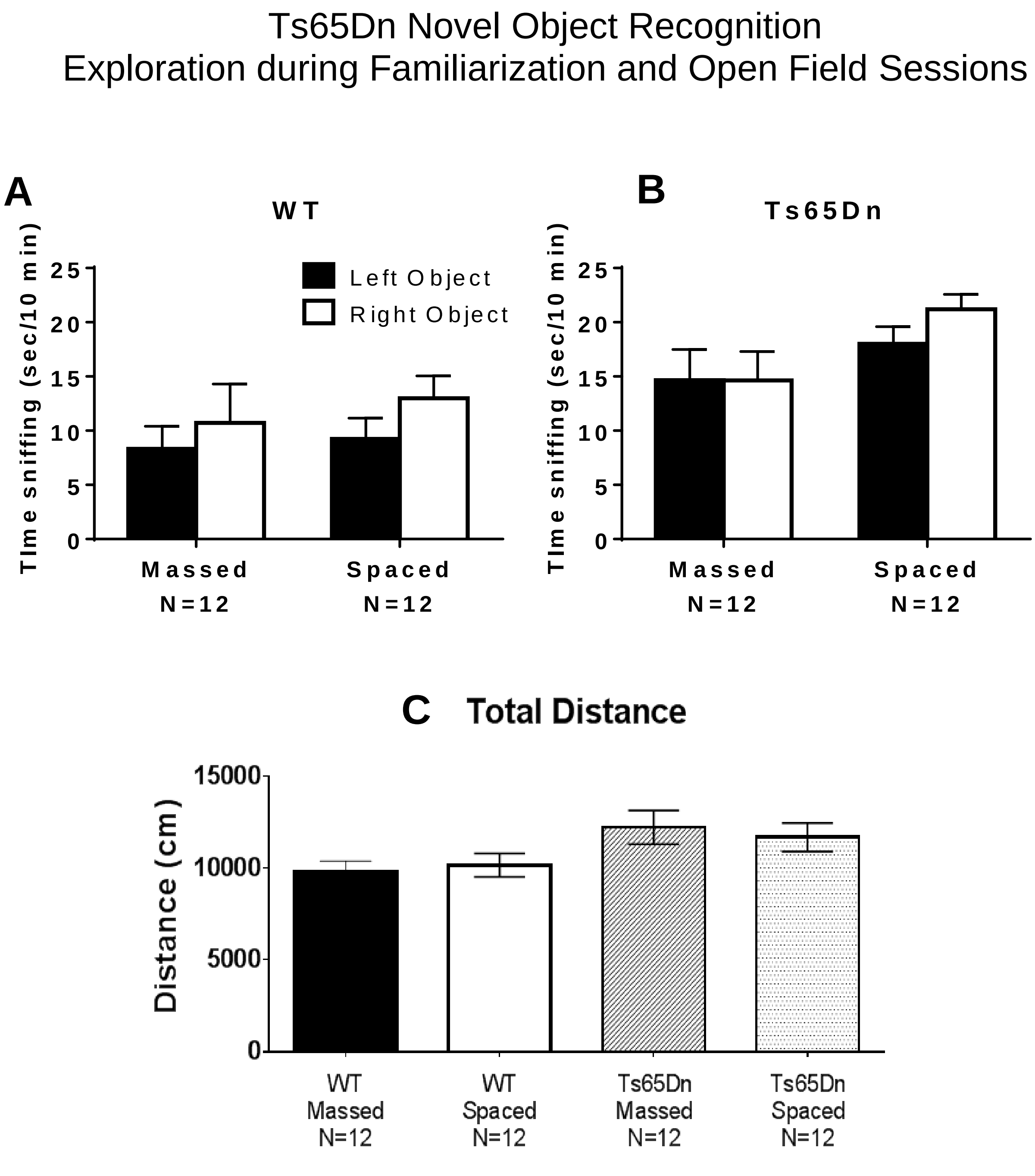

Supplementary Figure 2. Exploratory behaviors associated with novel object recognition were similar in WT and Ts65Dn mice, indicating no genotype differences in general locomotion or exploratory tendencies. No innate object bias was detected when the two identical novel objects were presented during the familiarization session, in either genotype or in either training condition group. (A) During the familiarization session administered 1 hour before the novel object recognition training session, WT did not display significant differences between time spent sniffing the two objects, identified as located in the right and left areas of the arena, in the group receiving a 10 minute massed familiarization session (massed: $t_{1,11} = 1.17$, NS), nor in the group receiving three 3.3 minute familiarization training trials, each separated by 3 minutes (spaced: $t_{1,10} = 2.18$, NS). (B) Ts65Dn did not display significant differences between time spent sniffing the two identical novel objects, located in the right and left areas of the arena, in the group receiving a 10 minute massed familiarization session (massed: $t_{1,11} = 0.007$, NS), nor in the group receiving three 3.3 minute familiarization training trials, each separated by 3 minutes: (spaced: $t_{1,11} = 1.86$, NS). (C) Open field exploratory locomotion, scored in the empty arena during a prior 30 minute habituation session. No significant differences were detected in total distance travelled across genotypes and training conditions (One-Way ANOVA $F_{3, 44} = 2.488$, $p=0.0728$, NS).

Supplemental Figure 3

Morris water maze swim speeds

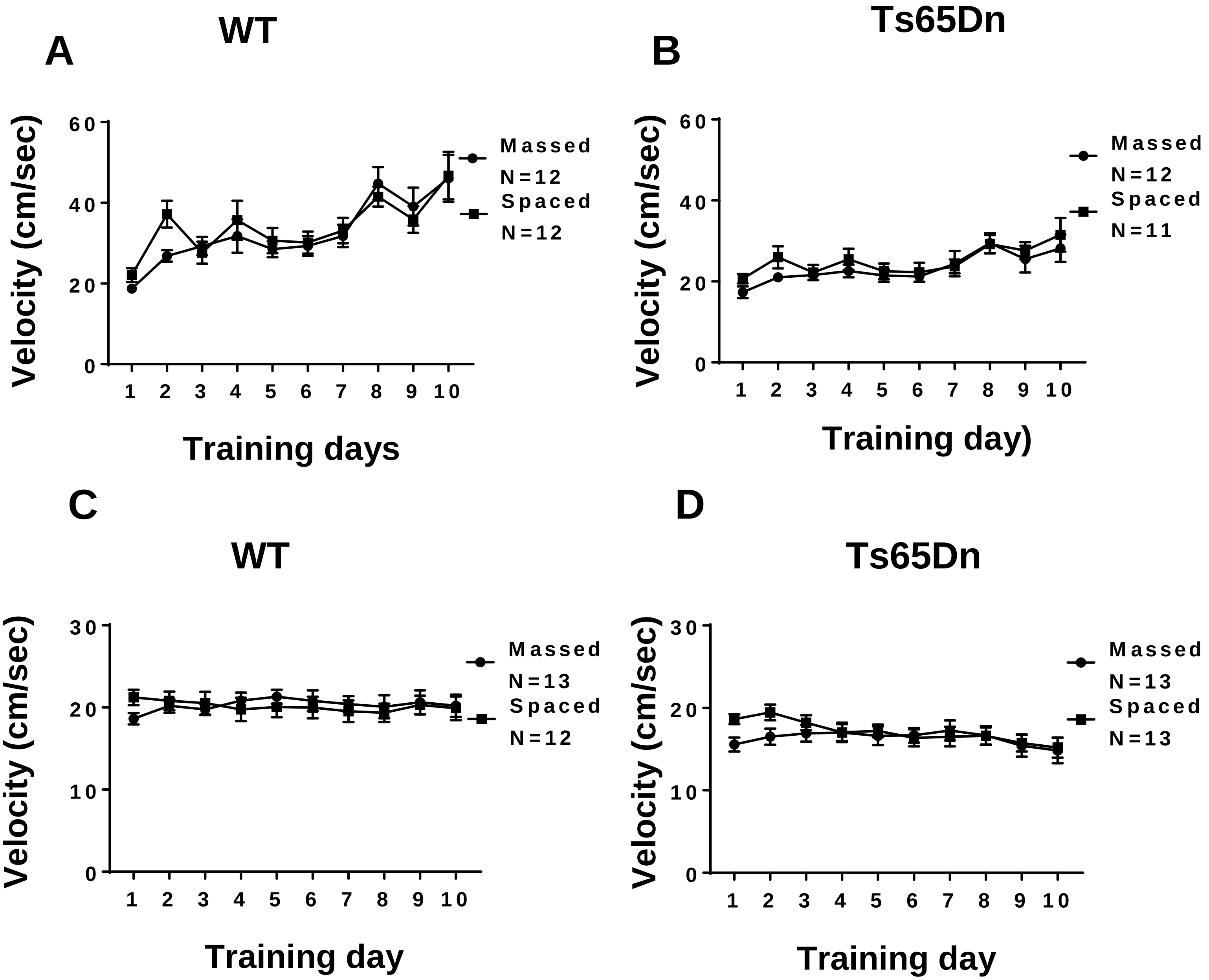

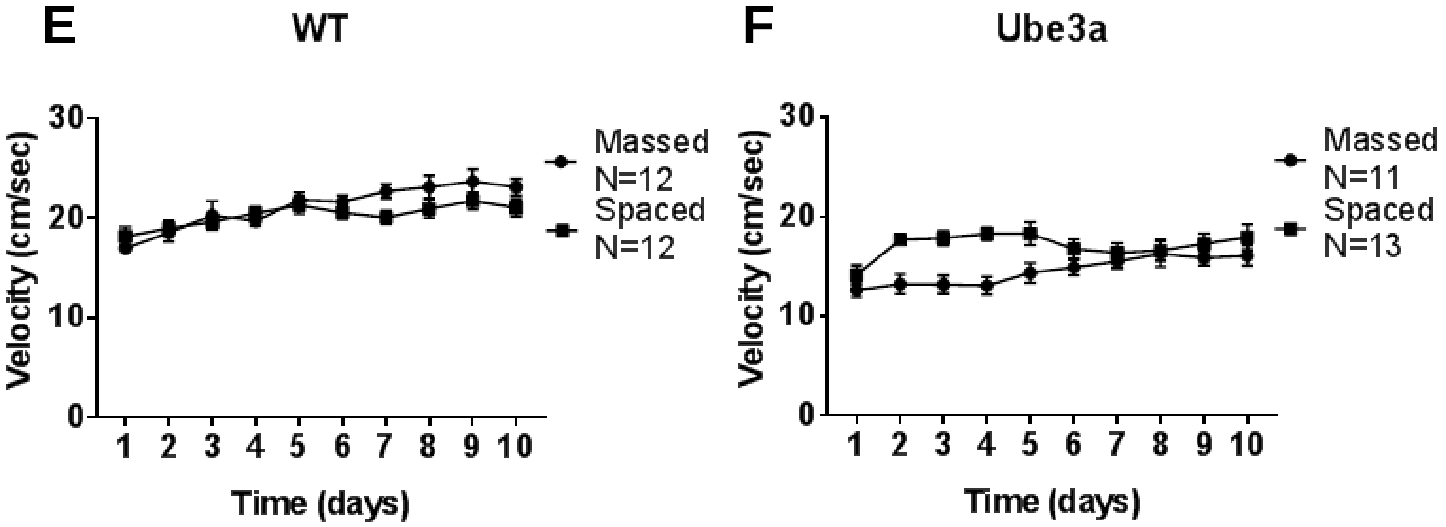

E
WT
Velocity (cm/sec)
30
20
10
0
1 2 3 4 5 6 7 8 9 10
Time (days)
Massed N=12
Spaced N=12
F
Ube3a
Velocity (cm/sec)
30
20
10
0
1 2 3 4 5 6 7 8 9 10
Time (days)
Massed N=11
Spaced N=13

Supplementary Figure 3. Swimming speed (velocity) was analyzed during Morris water maze training in two cohorts of Ts65Dn mice and in *Ube3a* mice during the massed versus spaced training regimes. (A,B) Cohort 1 Ts65Dn showed a genotype difference, ($F_{1,1} = 70.7$, $p<0.001$), with faster swimming by WT becoming apparent during later training days. (A) No difference in swim speeds was detected between WT mice trained with massed versus spaced trials ($F_{1,22} = 0.18$, NS). A significant effect of training day was detected in WT ($F_{9,198} = 16.7$, $p < 0.001$), but no significant interaction was detected between massed versus spaced x training day ($F_{9,198} = 1.19$, NS). (B) No difference in swim speeds was detected between Ts65Dn mice trained with massed versus spaced trials ($F_{1,21} = 0.601$, NS), indicating that better acquisition with spaced training was not due to faster swimming. A significant effect of training day was detected ($F_{9,189} = 8.541$, $p < 0.001$), but no significant interaction between massed versus spaced x training day ($F_{9,189} = 0.561$, NS). (C,D) Cohort 2 Ts65Dn showed an overall genotype difference, with WT swimming faster than Ts65Dn ($F_{1,1} = 35.8$, $p<0.001$), apparent during later training days. (C) No difference was detected in swim speeds between WT mice trained with massed versus spaced trials ($F_{1,23} = 0.01$, NS). No significant effect of training day on swim speed was detected in WT ($F_{9,207} = 0.44$, NS), and no significant interaction between massed versus spaced x training day was detected in WT ($F_{9,207} = 1.68$, NS). (D) No difference was detected in swim speeds between Ts65Dn mice trained with massed versus spaced trials ($F_{1,24} = 0.332$, NS), indicating that better acquisition with spaced training was not due to faster swimming. A significant effect of training day was detected in Ts65Dn ($F_{9,216} = 4.614$, $p <0.001$). A significant interaction between massed versus spaced x training day was detected in Ts65Dn ($F_{9,216} = 2.60$, $p < 0.01$. (E,F) *Ube3a* showed an overall genotype difference, with WT swimming faster than *Ube3a* ($F_{1,1} = 68.1$, $p < 0.001$). (E) No difference was detected in swim speeds in WT mice trained with massed versus spaced trials ($F_{1,22} = 1.404$, NS). A significant effect of training day was detected in WT ($F_{9,198} = 10.61$, $p < 0.001$), but no significant interaction between massed versus spaced x training day ($F_{9,198} = 1.822$, NS). (F) *Ube3a* mice trained with spaced trials swam faster than *Ube3a* trained with massed trials ($F_{1,122} = 7.75$, $p <0.05$), indicating that faster swimming during the first training days may have contributed to faster learning in *Ube3a* trained with spaced trials. A significant effect of training day was

detected ($F_{9,198} = 3.889$, $p < 0.001$), with a significant interaction between massed versus spaced x training day in *Ube3a* ($F_{9,198} = 3.098$, $p < 0.01$).
